# Surface dose variations in 6 and 10 MV flattened and flattening filter‐free (FFF) photon beams

**DOI:** 10.1120/jacmp.v17i5.6284

**Published:** 2016-09-08

**Authors:** Jason Cashmore

**Affiliations:** ^1^ Hall‐Edwards Radiotherapy Research Group, University Hospital Birmingham NHS Foundation Trust UK

**Keywords:** skin dose, filter free, unflattened, surface dose

## Abstract

As the use of linear accelerators operating in flattening filter‐free (FFF) modes becomes more widespread, it is important to have an understanding of the surface doses delivered to patients with these beams. Flattening filter removal alters the beam quality and relative contributions of low‐energy X‐rays and contamination electrons in the beam. Having dosimetric data to describe the surface dose and buildup regions under a range of conditions for FFF beams is important if clinical decisions are to be made. An Elekta Synergy linac with standard MLCi head has been commissioned to run at 6 MV and 10 MV running with the flattening filter in or out. In this linac the 6 MV FFF beam has been energy‐matched to the clinical beam on the central axis ($D_{10}$). The 10 MV beam energy has not been adjusted. The flattening filter in both cases is replaced by a thin (2 mm) stainless steel plate. A thin window parallel plate chamber has been used to measure a comprehensive set of surface dose data in these beams for variations in field size and SSD, and for the presence of attenuators (wedge, shadow tray, and treatment couch). Surface doses are generally higher in FFF beams for small field sizes and lower for large field sizes with a crossover at $10\times 10\;{\text{cm}}^{2}$ at 6 MV and $25\times 25\;{\text{cm}}^{2}$ at 10 MV. This trend is also seen in the presence of the wedge, shadow tray, and treatment couch. Only small differences (<0.5%) are seen between the beams on varying SSD. At both 6 and 10 MV the filter‐free beams show far less variation with field size than conventional beams. By removing the flattening filter, a source of contamination electrons is exchanged for a source of low‐energy photons (as these are no longer attenuated). In practice these two components almost balance out. No significant effects on surface dose are expected by the introduction of FFF delivery.

PACS number(s): 87.53.Bn, 87.55.ne, 87.56.bd

## I. INTRODUCTION

It is well known that megavoltage (MV) photon beams provide a skin‐sparing effect, but the actual magnitude of this can depend on a number of treatment parameters. Doses received by the basal skin layer can result in a range of problems from minor (such as erythema/epilation) to serious (desquamation/necrosis), depending on the doses received. At the same time it is also important that doses to targets close to the surface (such as for head and neck and breast treatments) are accurately known so that underdosage does not occur.

Changes in field size, source‐to‐surface distance (SSD) and beam energy all cause changes in surface dose. In particular, the introduction of materials into the beam line, such as shadow trays and patient support devices, can cause large increases in dose to the skin. A full knowledge and understanding of these doses is, therefore, necessary if clinical decisions are to be made.

Until recently, the flattening filter was generally considered to be a standard component in C‐arm linear accelerators, but with the introduction of accelerators, such as the Versa HD (Elekta AB, Stockholm, Sweden) and TrueBeam (Varian Medical Systems, Palo Alto, CA), the clinical use of flattening filter‐free (FFF) radiotherapy is increasing. Although surface doses have been studied under a range of conditions for conventional “flattened” beams,^1^, ^2^, ^3^, ^4^, ^5^, ^6^, ^7^ there is very little data for filter‐free beams. Several authors have reported simple surface dose vs. field size variations in FFF beams,^8^, ^9^, ^10^, ^11^ but data for changes in SSD and in the presence of absorbers, such as the shadow tray, wedge, and couch, are unavailable.

With this in mind, measurements of surface doses for FFF beams, and a comparison to standard flattened beams, are required to assess any potential changes in dose to the skin that may result from these new techniques.

The purpose of this investigation was to evaluate and compare trends in surface dose for 6 and 10 MV flattened and FFF beams under conditions routinely experienced in radiotherapy treatments. These conditions were:

- variations in field size (square fields, sizes 3×3 to $40\times 40\;{\text{cm}}^{2}$);
- variation in source‐surface‐distance (SSD) (55 to 140 cm); and
- the presence of:
- motorized wedge (0°–60°)
- block mounting tray
- carbon fiber treatment couch.

### A. Background

#### A.1 Skin doses

Skin doses arise from a combination of effects that will depend on the magnitude and relative contributions from contamination electrons, low‐energy photons, and backscattered radiation. Contamination electrons in particular can give rise to significant doses to the skin and will be influenced by several factors such as head design, beam energy, SSD, field size, or by the introduction of materials into the beam close to the patient. Skin doses can therefore vary significantly from one setup to another and can be a limiting factor in some treatments.^(1)^

Since the flattening filter is responsible for the majority of contamination electrons reaching the patient surface^(12)^ its removal is therefore likely to reduce this contribution. However, the filter also acts as a beam hardener, removing low‐energy photons from the spectrum. With the filter removed, this low‐energy component is allowed to pass through to the patient and will act to increase the surface dose. Whether this results in higher or lower skin doses in clinical situations is, therefore, of interest.

Motorized wedges and Perspex (Mitsubishi Rayon Lucite Group Ltd., Southampton, UK) shadow trays (blocking trays) are in common use in conventional radiotherapy and are known to effect surface doses. Although FFF is associated with small‐field and IMRT techniques, their use in 3D CRT has been demonstrated;^13^, ^14^ therefore, FFF data have also been measured for the motorized wedge and shadow tray. Doses measured under these conditions can also be a good indicator of the relative levels of dose from low‐energy photons and from contamination electrons (although no attempt has been made to separate these two components here).

#### A.2 Dose measurements

In the buildup region of high‐energy photon beams electronic equilibrium does not exist, therefore percentage depth ionization (PDI) and percentage depth dose (PDD) are not equal. Under these conditions, electronic fluence perturbations in the air‐sensitive volume of the cavity cause ionization chambers to exhibit an “overresponse,” and determining PDD from PDI measurements is challenging. This effect will occur for measurements carried out in the dose buildup region (and in the transition zones between different media), with a magnitude depending on the geometrical characteristics of the chamber. Any overresponse will be greatest at the surface and decrease as dose maximum ($d_{\text{max}}$) is approached, where electronic equilibrium is restored.

In these circumstances, it is generally accepted that extrapolation chambers provide the most accurate means of measurement. These chambers, however, are unavailable in most departments and are also very time‐consuming to use; therefore parallel plate chambers are routinely used to measure surface and buildup doses, and corrections must be applied to convert ionization to dose.

The overresponse of the chamber has been shown to be mainly due to electron in‐scattering from the side walls, which is determined by the width of the guard ring.^15^, ^16^ Perturbation factors will therefore depend on the dimensions of the chamber, and several methods have been introduced to correct for the perturbation effects seen when using parallel plate chambers in the buildup region of high‐energy photon beams. These are based either on correction based methods from extrapolation chamber measurements, or by direct derivation of individual perturbation factors.

In the first method, the chamber reading is corrected by a subtractive factor that depends on the beam energy, chamber geometry, and depth of measurement.^15^, ^16^, ^17^, ^18^ These factors are essentially independent of field size, but increase with decreasing energy.

In the second method, perturbation factors are derived explicitly. For parallel‐plate chambers the PDD at depth d can be expressed as

(1) $$\text{PDD}(d)=\text{PDI}(d)\frac{{\left[{\left(\frac{\bar{L}}{\rho }\right)}_{\text{air}}^{\text{med}}P_{fl}P_{\text{wall}}P_{\text{cel}}\right]}_{d}}{{\left[{\left(\frac{\bar{L}}{\rho }\right)}_{\text{air}}^{\text{med}}P_{fl}P_{\text{wall}}P_{\text{cel}}\right]}_{d_{\max}}}$$

where ${\left(\bar{L_{\Delta }}/\rho \right)}_{\text{air}}^{\text{med}}$ is the mean restricted collision‐stopping power from medium to air and $P_{fl},P_{\text{wall}}$, and $P_{\text{cel}l}$ are perturbation factors for the fluence, wall, and central electrode, respectively.^(19)^ When $d>d_{\text{max}}$ these factors do not change and can be considered as invariant; the dose ratio is then equal to the ionization ratio (PDD=PDI). In the buildup region however, perturbations in the electron fluence cause these chambers to overrespond so that PDI>PDD. Since perturbation factors are known to have a strong dependence on chamber design, depth of measurement and beam energy, $P_{fl},P_{\text{wall}},P_{\text{cel}l}$ and ${\left(\bar{L_{\Delta }}/\rho \right)}_{\text{air}}^{\text{med}}$ must be independently evaluated.

Recent studies have shown that the product $P_{fl},P_{\text{wall}},P_{\text{cel}l}$ changes by less than 1% from the surface to $d_{\text{max}}$ ^20^, ^21^ and that ${\left(\bar{L_{\Delta }}/\rho \right)}_{\text{air}}^{\text{med}}$ changes by less than 4% for beams up to 18 MV. The maximum deviation occurs at the phantom surface and will be less than 5%, reducing to 0% at $d_{\text{max}}$.

For certain ion chambers and beam energies some of these factors have been evaluated.^(22)^ The presence of beam modifiers such as the wedge can change the photon beam spectrum appreciably, so likewise removal of the flattening filter and associated changes in the beam spectrum are therefore likely to cause significant changes to this. For FFF beams in general these are unknown, and an independent set of overresponse factors would be required for more accurate prediction.

To avoid the improper use of unknown correction factors for FFF beams, the effective point of measurement method as described McEwen et al.^(21)^ has been employed to calculate dose in the buildup region, accepting the limitations of this technique.

## II. MATERIALS AND METHODS

### A. Linear accelerator

An Elekta Synergy linear accelerator (Elekta AB, Stockholm, Sweden) with a standard 80‐leaf MLCi head has been used for measurements. The linac is fitted with a motorized wedge (nominal 60° wedge angle) and an iBEAM evo carbon fiber couchtop, both of which were assessed.

The flattening filter is replaced with a 2 mm stainless steel plate that is used to shield out contamination electrons from the primary collimator, and to provide buildup into the ionization chamber. Without this plate the signals in the chamber are too low to accurately control the beam energy and steering servos.

Removing the flattening filter changes the energy spectrum of the beam since lower energy photons are no longer removed from the beam. This results in FFF beams having “softer” beam spectra than their beam‐hardened counterparts. The 6 MV beam can be energy matched to the conventional beam by adjusting the beam running parameters. In this way the depth doses at 10 cm depth, $D_{10}$ (67.5%) and the quality index QI (${\text{TPR}}_{20/10}$) (0.675) for a $10\times 10\;{\text{cm}}^{2}$ field can be maintained. FF removal also affects the variation in the energy spectrum across the beam (making it much more uniform) but energy matching across the beam is not possible. In contrast, and in following with other work^10^, ^23^ the 10 MV beam has not been adjusted. The ${\text{TPR}}_{20/10}$ for the 10 MV and 10 MV FFF beams are 0.737 and 0.716, respectively.

### B. Experimental setup

**Figure 1:**
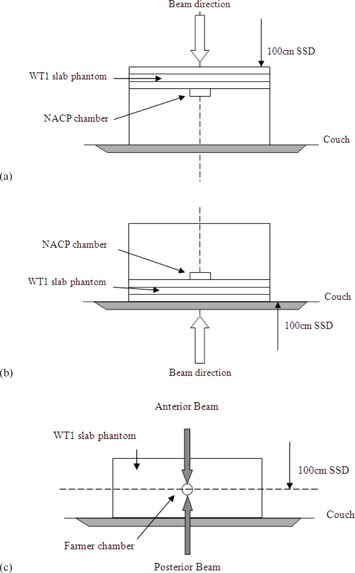
Experimental setup for (a) surface dose and buildup, (b) surface dose and buildup through the couch, (c) couch transmission factors.

Measurements were taken in a phantom composed of $30\times 30\;{\text{cm}}^{2}$ Solid Water slabs (Radiation Physics, St. Bartholomew's Hospital, London) of varying thickness, with 15 cm of backscatter material to ensure full phantom scatter conditions. Further sheets of Solid Water were added to take readings in the buildup region (maintaining SSD) (Fig. 1(a)). Doses were measured with an NACP‐02 (Scanditronix, IBA Dosimetry, Schwarzenbruck, Germany) parallel plate ionization chamber. The NACP chamber has a “coin‐shaped” sensitive volume with a diameter of 10 mm, a height of 2 mm and a guard ring of 3 mm in width.^(24)^ In the literature a guard ring of at least 5 mm is recommended for measuring skin doses,^(16)^ but both Carl and Vestergaard^(1)^ and McEwen et al.^(21)^ have investigated its use and found it to be suitable.

The chamber was embedded in the phantom such that the entrance window of the chamber was flush with the surface with the central axis perpendicular to this. Doses at depth were measured by adding layers of phantom material while maintaining the SSD at 100 cm to the top of the phantom (Fig. 1(a)). The effective point of measurement itself was taken as the inside of the entrance window which, for the NACP‐02 chamber (with a composite window of 0.1 mm Mylar and 0.5 mm graphite) is equivalent to 1 mm of water. Surface dose measurements therefore represent a measurement depth of 1 mm, and all results are plotted relative to the dose measured at $d_{\text{max}}$ (15 mm 6 MV, 22 mm 10 MV) for the same field size. Surface dose readings are therefore reported as relative surface dose (RSD) where $\text{RSD}=d_{\text{surface}}/d_{\text{max}}$.

Buildup curves through the carbon fiber couch we measured according to the setup shown in Fig. 1(b), with the gantry rotated to 180° to deliver radiation through the couch. Couch transmission factors were measured using a Farmer‐type chamber (NE2577 Nuclear Enterprises Ltd. (now supplied by Qados, Sandhurst, UK)) set at isocenter within 10 cm of WT1 (Fig. 1(c)). Couch factors are expressed as the ratio of the posterior to anterior electrometer readings.

Polarity corrections in X‐ray beams are generally small, but can become significant for parallel plate chambers, especially in the buildup region.^(25)^ It is expected that the largest differences will occur at the surface, and that this will reduce as $d_{\text{max}}$ is approached. To estimate polarity effects buildup curves were measured at ±200V over the buildup region, representing the full range of contamination conditions at 6 and 10 MV.

## III. RESULTS

### A. Polarity and ion recombination effects

**Figure 2:**
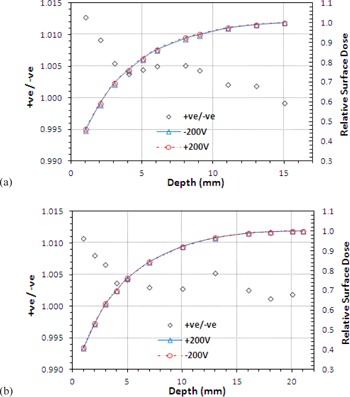
Relative surface dose as a function of depth for +ve and ‐ve polarization voltages for (a) 6 MV and (b) 10 MV photons. The +ve / ‐ve ratio shows the increasing disequilibrium towards the surface.

Surface doses were measured at positive and negative polarity (±200V) under representative high and low conditions (between surface and $d_{\text{max}}$). Figure 2 shows these buildup curves for both 6 and 10 MV FFF beams (for a $10\times 10\;{\text{cm}}^{2}$ field), and the ratio of the readings (+ve/‐ve) vs. depth.

As expected, the correction becomes larger as the measurement point moves closer to the surface and was found to differ by <1.3% (1.3% at 6 MV, 1.1% at 10 MV) and fall to unity at $d_{\text{max}}$. This was considered to be of small enough magnitude to disregard as the main purpose of this paper is to compare surface doses and evaluate trends. Readings were therefore taken at −200V only for the remainder of the study.

Ion recombination ($P_{\text{ion}}$) is known to have a greater effect in FFF beams due to the increase in dose per pulse (DPP),^26^, ^27^ and will vary with both depth and energy. As relative readings are used throughout the study the RSD remains relatively unaffected, with deviations estimated to be <0.5% for the cases studied. $P_{\text{ion}}$ corrections have therefore not been included.

### B. Field size variation

**Figure 3:**
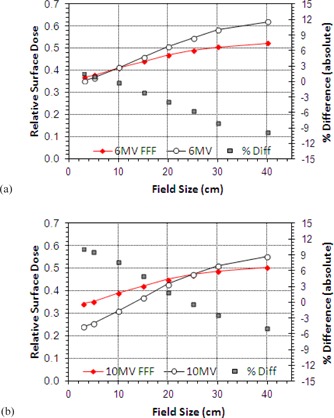
Variation of surface dose with field size for Flattened and FFF beams for jaw settings of 3×3 to $40\times 40\;{\text{cm}}^{2}$: (a) 6 MV, (b) 10 MV.

Surface doses are seen to increase almost linearly with increasing field size, albeit at a shallower incline for FFF beams than for the conventional (Fig. 3(a)). At 6 MV the unflattened beam shows a slight increase in RSD at smaller field sizes (+3.4% at $3\times 3\;{\text{cm}}^{2}$) and decrease at larger (−7.1% at $40\times 40\;{\text{cm}}^{2}$) with equivalency at $10\times 10\;{\text{cm}}^{2}$.

At 10 MV there is again far less variation in the RSD with field size for the FFF beams, but the conventional beam generally exhibits lower surface doses (Fig. 3(b)). At small field sizes the unmatched 10 MV FFF shows an increase of 10% in absolute terms over the conventional 10 MV beam; in contrast, at the largest field size the FFF beam shows a 5% decrease. The surface doses are of equivalent magnitude at a field size of $25\times 25\;{\text{cm}}^{2}$.

For the 6 MV beam, there is a variation in RSD of 26.7% (absolute) in changing from a 3×3 to $40\times 40\;{\text{cm}}^{2}$ field (0.353 to 0.620) compared to 16.2% (0.387 to 0.549) for the FFF equivalent. At 10 MV these values are 31.0% for 10 MV (0.242 to 0.552) and 16.0% for 10 MVFFF (0.344 to 0.504).

The variation of surface dose with FS is very similar for the 6 and 10 MV FFF beams showing only minor differences with field size ($0.44\%\;{\text{cm}}^{-1}$ FFF at 6 MV and $0.43\%\;{\text{cm}}^{-1}$ at 10 MV). For conventional operation these gradients are $0.72\%\;{\text{cm}}^{-1}$ at 6 MV and $0.84\%\;{\text{cm}}^{-1}$ at 10 MV.

### C. Wedge

**Figure 4:**
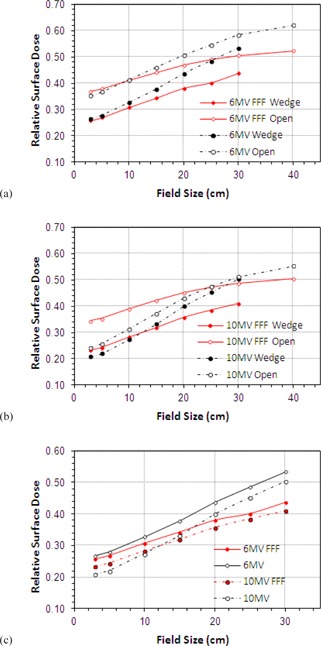
Variation of RSD with field size for wedged beams: (a) 6 MV FFF and conventional open and wedged beams, (b) variation at 10 MV, (c) wedge only data for 6 and 10 MV.

The skin dose for wedged field increases as field size is increased in a similar manner to the skin dose for open fields. However, with the wedge in place surface doses at both 6 and 10 MV are seen to be generally lower for FFF beams. This is true at all field sizes for 6 MV (Fig. 4(a)) and those beyond 12×12cm for 10 MV (Fig. 4(b)). The slope of the curves is also much shallower for the unflattened beams. This is demonstrated clearly in Fig. 4(c), where wedge‐only data are plotted for each beam. The gradient of these lines is $0.66\%\;{\text{cm}}^{-1}$ vs. $0.99\%\;{\text{cm}}^{-1}$ at 6 MV and $0.65\%\;{\text{cm}}^{-1}$ and $1.09\%\;{\text{cm}}^{-1}$ at 10 MV, again demonstrating much less variation with field size for filter‐free operation.

**Figure 5:**
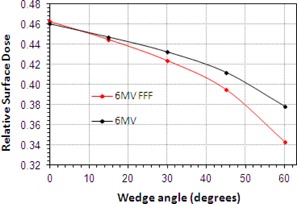
RSD variation with wedge angle for motorized wedge, 6 MV. Field size is $15\times 15\;{\text{cm}}^{2}$.

Skin doses for wedge angles of 15°, 30°, 45°, and 60° were also measured for the motorized wedge at 6 MV only (Fig. 5). Since the wedge is shown to reduce RSD the greatest difference is observed when the field is fully wedged.

### D. SSD variation

**Figure 6:**
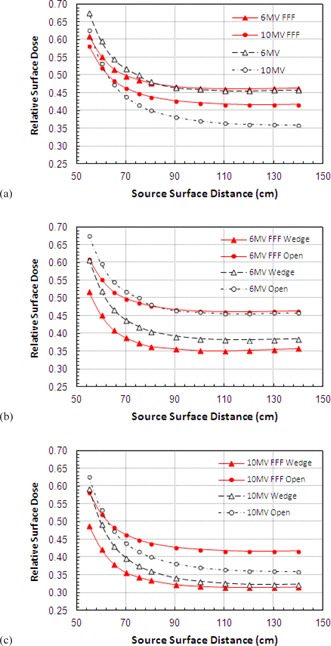
Effect of changes in source to surface distance on surface dose for 6 MV and 10 MV beams for open and wedged beams ($15\times 15\;{\text{cm}}^{2}$).

At 6 MV there is little difference between the FFF and conventional beams, except near the treatment head where the FFF beams show a reduction in surface dose of up to 5% (Fig. 6(a)). Increasing the beam energy to 10 MV shows the typical reduction in surface dose. The FFF beam again shows reduced doses at the shortest SSDs, but this quickly reverses with the conventional 10 MV beams exhibiting a marked reduction in dose beyond 60 cm SSD.

RSD is very stable between 90 and 140 cm SSD, then shows a sharp rise for measurements closer to the treatment head. Again, there is a reduction seen in the variation in FFF beams compared to that for conventional beams.

SSD variations were also investigated with the wedge in place. At both 6 (Fig. 6(b)) and 10 MV (Fig. 6(c)) a larger dose difference is seen between FFF wedge and open data than is seen for the conventional beams. The highest RSD is seen for 10 MV FFF beams, and lowest for the 10 MV wedge. A 10% reduction in RSD is seen for 10 MV FFF compared to only 4% for 10 MV. At 6 MV these figure are 11% vs. 7%.

### E. Shadow tray

**Figure 7:**
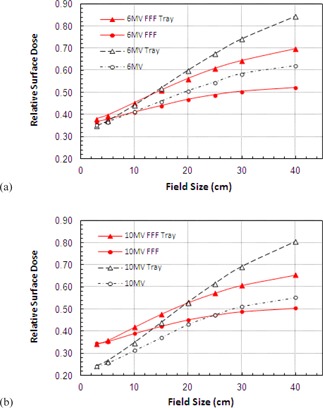
Variation of RSD when passing through a Perspex shadow tray for (a) 6 MV and (b) 10 MV.

Inevitably, the insertion of an acrylic shadow tray (10 mm thick acrylic (polymethyl meth‐acrylate)) into the beam causes an increase in the observed surface dose. At 6 MV the smallest field sizes exhibit very similar readings, but these rapidly diverge as field size increases with the FFF beam having a much shallower slope (Fig. 7(a)). For a $40\times 40\;{\text{cm}}^{2}$ field the difference decreases by 12% for the FFF beam. For the 10 MV beams the slope is once again much shallower for the FFF energy (Fig. 7(b)). There is a switch‐over from higher to lower surface doses at around $20\times 20\;{\text{cm}}^{2}$ with doses being approximately 10% higher for $3\times 3\;{\text{cm}}^{2}$ and 15% lower for $40\times 40\;{\text{cm}}^{2}$.

At 10 MV a reduced variation with field size is again prevalent with a gradient reduction for FFF. The slope of FFF curves is calculated to be $0.85\%\;{\text{cm}}^{-1}$ FFF vs. $1.33\%\;{\text{cm}}^{-1}$ at 6 MV and $0.85\%\;{\text{cm}}^{-1}$ vs. $1.52\%\;{\text{cm}}^{-1}$ at 10 MV.

### F. Carbon fiber couch

**Figure 8:**
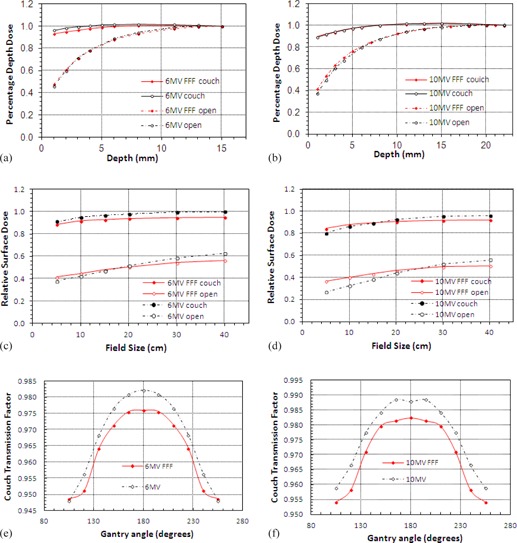
Buildup curves for (a) 6 MV and (b) 10 MV with and without the treatment couch intersecting the beam. Relative surface dose vs. field size for (c) 6 MV and (d) 10 MV. Couch transmission factors vs. gantry angle (e), (f).

Figures 8 (a) and 8(b) show the buildup characteristics when passing through the couch. The curves for both 6 and 10 MV show very little difference between FFF and conventional beams. At 6 MV there is a slight decrease for FFF (∼3%) at the surface, but not of sufficient magnitude to be of clinical significance. As the field size is varied from 5×5 to $40\times 40\;{\text{cm}}^{2}$, the difference is slightly exaggerated (up to 6%) (Figs. 8(c) and 8(8)).

Couch transmission factors follow a similar pattern between FFF and conventional energies with the FFF beams showing a greater factor at both energies, 0.976 vs. 0.982 for 6 MV and 0.982 vs. 0.988 for 10 MV. The variation of couch transmission factor with changing gantry angle is shown in Figs. 8(e) and 8(8) for 6 and 10 MV, respectively.

## IV. DISCUSSION AND CONCLUSIONS

By passing through the flattening filter, conventional beams exhibit a hardened beam spectrum, so tend to contain a low amount of low‐energy X‐rays, which will lead to lower surface doses, but high amounts of contamination electrons, raising surface doses. FFF beams on the other hand contain larger amounts of low‐energy photons since these are no longer filtered out by the flattening filter, but lower amounts of contamination electrons, reversing the situation seen in the conventional filtered beam. In practice, it seems that these effects almost cancel each other out so that the overall magnitude of surface doses from FFF beams is not too dissimilar from those seen in the original beams.

The increase in surface dose with increasing field size is a well‐known effect and it is electron contamination that gives rise to most of this variation,^(28)^ so the overriding effect of filter removal is a reduction in the variation of surface doses with field size. Here, the reduction in electron contamination means that the FFF beams show much less variation with changes in field size compared to filtered beams. In general surface doses in FFF are higher for small field sizes and lower for larger field sizes with the crossover at $10\times 10\;{\text{cm}}^{2}$ for 6 MV and at $25\times 25\;{\text{cm}}^{2}$ for 10 MV.

Kragl et al.^(10)^ observed a similar variation in surface dose with field size for an energy‐matched Elekta linac (42.3% at $5\times 5\;{\text{cm}}^{2}$, 56.5% at $30\times 30\;{\text{cm}}^{2}$ compared to 39.7% and 53.1% seen here). These readings are 2% to 3% higher than those seen in this study but could be explained by the use of a 6 mm Cu plate in earlier versions of the filter plate, instead of the 2 mm stainless steel used in the final, clinical configuration. The Kragl study also reported data for a nonenergy‐matched Elekta linac at 6 MV, reporting surface doses of 47.1% at $5\times 5\;{\text{cm}}^{2}$ and 61.6% at $30\times 30\;{\text{cm}}^{2}$, and increase of approximately 5% over operation in the energy‐matched linac. In the unmatched machine, the average energy is reduced to around 5 MV by filter removal, so this increase in surface doses (by approx. 5%) can be explained by the change in beam quality.

A similar situation is seen for the Varian TrueBeam accelerator where the beam energy is not adjusted and FFF doses are seen to be higher at all field sizes for both 6 and 10 MV compared to operation with the filter in place.^8^, ^11^ Surface doses are strongly dependent on beam energy, so by not altering the beam energy to match, maintaining ${\text{TPR}}_{20/10}$ on the central axis, surface doses will increase.

Materials in the path of the beam can both generate electrons and absorb them from further upstream. Since it is relatively thin and of low‐Z material, the tray generates more electrons than it stops, and these pass through to reach the patient, raising skin doses. As the field size increases more electrons are emitted from the shadow tray, therefore higher doses are expected for larger field sizes compared to open fields.

Fontenla et al.^(29)^ showed an increase in the skin dose of approximately 16% when a tray was added into a $25\times 25\;{\text{cm}}^{2}$ field at 6 MV. Our data show an increase of 13% for the conventional beams and 9.5% for the 6 MV FFF. At 10 MV these figures are 14.2 and 10%, respectively.

The wedge is of sufficient thickness to absorb all of the contamination electrons rising from the head of the linac,^(30)^ but surface doses will be influenced by electrons generated within the wedge. These are produced in a thin layer close to the exit surface, equivalent in thickness to the range of the secondary electrons.

Regardless of beam energy, the presence of the wedge is seen to reduce surface doses by up to 10% for larger field sizes. Compared to conventional fields the FFF beams again show a reduction in RSD across all field sizes with the slope of the curve being much shallower for FFF operation, and filter removal is seen to have a much larger effect on the 10 MV FFF beam than the 10 MV (10% at 10 MV FFF compared to 4% for 10 MV). Considering the blocking tray, wedged fields, and open fields together, the overall reduction in the slope of the curves with field size is seen to be reduced by 36.1% at 6 MV and 44.4% at 10 MV.

Considering the variation of RSD with SSD at 6 MV, it seems that this remains very stable at SSDs beyond 90 cm. At shorter SSDs there is a significant rise in surface dose for both beams, but to a greater extent for the conventional 6 MV. This again indicates the presence of a greater number of contamination electrons in the filtered beam, which are gradually absorbed by the air column as SSD increases. At 10 MV the surface dose is generally higher for the FFF beams at all but the shortest SSDs, but the variation is much reduced for FFF operation. Again an approximate 10% reduction in RSD is seen on filter removal at both beam energies with the wedge in place, and this is consistent across all SSDs.

Increasing the beam energy is known to reduce surface dose, so even though 10 MV FFF doses are seen to be slightly higher than for standard 10 MV operation, they are still lower than those seen at 6 MV. At 10 MV differences in surface dose may be due to the lower effective energy of the beam compared to the standard, as in this study the 10 MV FFF beam has not been energy matched. However, the small variation seen between 6 MV FFF and 10 MV FFF over a range of conditions indicates that a change in beam energy to match the 10 MV might have little overall effect.

Several authors have studied the effects of carbon fiber couchtops on surface dose.^6^, ^31^, ^32^, ^33^ The proximity of the patient to the couch means that the skin sparing effect is almost completely lost and surface doses increase to 85%–95% of that seen at $d_{\text{max}}$, and for FFF beams a similar pattern is observed. At 6 MV a very slight reduction in %DD is seen for FFF operation, and at 10 MV the buildup curves are almost identical. The variation of RSD with field size is lower for 6 MV FFF over all field sizes, and lower at 10 MV FFF beyond $15\times 15\;{\text{cm}}^{2}$.

Since the unflattened beams contain more low‐energy photons than conventional beams the transmission factor of the couch is greater for both FFF energies (by approx. 0.5%), showing a similar variation with gantry angle to that observed for normal use.

Removal of the flattening filter has been shown to have many potential benefits over conventionally filtered beams for the delivery of treatments techniques such as (but not limited to) SRT, SBRT, and IMRT. The present study indicates that the surface doses from these beams are very similar to those experienced for conventional flattened beams, and are therefore unlikely to cause concern in a clinical setting. However, as variations in electron contamination with SSD, or from the introduction of wedges, trays and couches are not taken into account in most treatment planning systems,^(27)^ for the moment it is possible that the use of FFF beams may help to reduce the uncertainty of dose calculations in the buildup region.

## COPYRIGHT

This work is licensed under a Creative Commons Attribution 3.0 Unported License.
